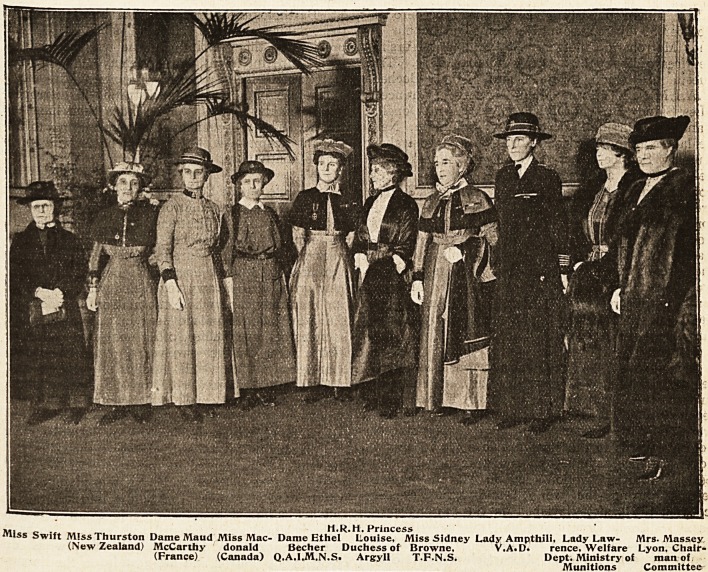# Military and Overseas Matrons-In-Chief

**Published:** 1918-11-09

**Authors:** 


					November 9:, 1918. THE HOSPITAL 117
MILITARY AND OVERSEAS MATRONS-IN-CHIEF.
,,, . H.R.H. Princess
"liss Swift M!ss Thurston Dame Maud Miss Mac- Dame Ethel Louise. Miss Sidney Lady Ampthili. Lady Law- Mrs. Massey
(New Zealand) McCarthy donald Becher Duchess of Browne, V.A.D. rence. Welfare Lyon, Chair-
(France) (Canada) Q.A.1.M.N.S> Argyll T.F.N.S. Dept. Ministry of man of
Munitions Committee

				

## Figures and Tables

**Figure f1:**